# Malarial Paraplegia Relieved by Quinine

**Published:** 1886-09

**Authors:** C. W. Wilkinson

**Affiliations:** Surgeon in charge of St. Mary’s Hospital, Galveston, Tex.


### HOSPITAL PRACTICE,

### MALARIAL PARAPLEGIA — RELIEVED WITH SULPHATE OF QUINIA.

*Reported for Daniel's Texas Medical Journal, by C. W. Wilkinson, M. D., Surgeon in charge of St. Mary's Hospital, Galveston, Tex.*

### HISTORY OF CASES.

John P. Wurts, aged 23 years, was struck with paralysis on the 12th day of June, 1886, at the town of Abilene, Texas. At that time he had been away from Galveston, his old home, about 13 months, having been most of the time at Corsicana, Dallas and Fort Worth, and all the while living in good health.

On the 6th day of June he was caught in a severe hail storm, and, as he said, "took cold." On the next day, 7th, he was seized with a "chill and fever," which lasted until in the night; on the 8th he was well, and on the 9th he had another "chill and fever," which lasted into the night. He says "my fevers were broken up with quinine, and I went then to Abilene. On the morning of the 12th I was in a barber's chair, not feeling well, and getting worse, I left the shop to go to my boarding house. I could hardly drag my legs after me, and felt very weak. My condition gradually grew worse, till on the 14th I could not move my legs and was laid up in bed." On the 23d of June he arrived in Galveston and was taken to St.

Mary's hospital in the following condition: Lower limbs completely paralysed as to motion, but highly alive to sensation. Could bear no pressure upon either limb without great pain.

Bowels and bladder, both paralyzed; one unable to retain and the other to expel its contents; upper extremities healthy; surface pale; patient entirely helpless, can not move a muscle of his lower extremities; no fever.

In this condition of paraplegia he remained several days, gradually growing worse, until on the evening of July the 4th, he was seized with a convulsion of an epileptiform character. Up to this time he had been taking iodide of potassium, 40 grains a day, under the impression that he had been the victim of a former syphilitic attack. The plan of treatment pursued, however, did not im prove the patient, as demonstrated by the sequel. His convulsive attack was treated by morphine and sulphuric ether, administered hypodermically, but so prostrated was the case for several hours afterwards, that an unfavorable prognosis was given without hesitation.

The patient, through the treatment he received at the time, did not succumb to his severe attack, but remained in a prostrated condition for several days afterwards, supported mainly on beef tea and stimulants. He now began to complain of pains occurring in his lower limbs every evening, which would last, usually, several hours before leaving, and which annoyed him very considerably.

Upon the ground that a periodical disorder demanded an antiperiodic remedy, I ordered 15 grains of sulphate of quinia to be given Mr. Wurtz before his pain hour, every evening. The effect of the remedy was very promptly and markedly demonstrated; the pains recurred very slightly after the first administration of the drug and *never returned at all after the second*, nor did his improvement cease with the subsidence of his pains. Motion became restored in his limbs, and within a week from the beginning of the quinine treatment, the patient was out of bed, and walking all about the hospital. This patient has continued to improve. About the 20th day of July, I suspended the quinine treatment, with a view of testing the capacity of the patient to withstand the remedy. He required further treatment, however, as towards the 27th, he began to grow weary in his limbs, and was unable to walk about as freely as during the week before; moreever a little pain returned in his thigh, and I resorted to quinine in 20 grain doses again. At date of writing, August 28, he is on his feet, and seemingly in splendid health.

